# Elevated H3K27me3 levels sensitize osteosarcoma to cisplatin

**DOI:** 10.1186/s13148-018-0605-x

**Published:** 2019-01-16

**Authors:** Chao He, Jian Sun, Chao Liu, Yuhang Jiang, Yongqiang Hao

**Affiliations:** 1 Shanghai Key Laboratory of Orthopedic Implants, Department of Orthopedic Surgery, Shanghai Ninth People’s Hospital, Shanghai Jiao Tong University School of Medicine, Shanghai, 200011 China; 2 Department of Emergency, Shanghai Jiao Tong University Affiliated Sixth People’s Hospital, Shanghai, 200233 China; 3 Department of Oromaxillofacial Head and Neck Oncology, Shanghai Ninth People’s Hospital, College of Stomatology, Shanghai Jiao Tong University School of Medicine, Shanghai, 200011 China

**Keywords:** Osteosarcoma, H3 lysine 27 trimethylation (H3K27me3), KDM6A, KDM6B, Chemoresistance, Apoptosis

## Abstract

**Background:**

In osteosarcoma (OS), chemotherapy resistance has become one of the greatest issues leading to high mortality among patients. However, the mechanisms of drug resistance remain elusive, limiting therapeutic efficacy. Here, we set out to explore the relationship between dynamic histone changes and the efficacy of cisplatin against OS.

**Results:**

First, we found two histone demethylases associated with histone H3 lysine 27 trimethylation (H3K27me3) demethylation, KDM6A, and KDM6B that were upregulated after cisplatin treatment. Consistent with the clinical data, cisplatin-resistant OS specimens showed lower H3K27me3 levels than sensitive specimens. Then, we evaluated the effects of H3K27me3 alteration on OS chemosensitivity. In vitro inhibition of the histone methyltransferase EZH2 in OS cells decreased H3K27me3 levels and led to cisplatin resistance. Conversely, inhibition of the demethylases KDM6A and KDM6B increased H3K27me3 levels in OS and reversed cisplatin resistance in vitro and in vivo. Mechanistically, with the help of RNA sequencing (RNAseq), we found that PRKCA and MCL1 directly participated in the process by altering H3K27me3 on their gene loci, ultimately inactivating RAF/ERK/MAPK cascades and decreasing phosphorylation of BCL2.

**Conclusions:**

Our study reveals a new epigenetic mechanism of OS resistance and indicates that elevated H3K27me3 levels can sensitize OS to cisplatin, suggesting a promising new strategy for the treatment of OS.

**Electronic supplementary material:**

The online version of this article (10.1186/s13148-018-0605-x) contains supplementary material, which is available to authorized users.

## Background

Osteosarcoma (OS) is the most common primary malignant bone tumor worldwide, with an incidence of approximately one to three cases per million people per year; the male to female ratio is approximately 1.22:1 [1], and children/adolescents and those over the age of 75 with Paget’s disease or other bony lesions are the two susceptible populations [2, 3]. The femur (40%), tibia (20%), humerus (10%), and pelvis (8%) together account for almost 80% of the bones involved in OS based on reported cases; the other involved bones include the fibula, jaw, vertebrae, radius, ulna, and ribs [3]. OS patients with metastasis and recurrence have a poor prognosis that is partly attributed to chemoresistance [4, 5]. Although several mechanisms have been reported to mediate chemoresistance and some chemoresistance-reversing agents have even been developed, the mechanisms remain unclear, and almost no substantial survival benefits are conferred by administration of the therapeutic agents [6]. Above all, further exploration of the mechanism of chemoresistance and development of new strategies to reverse chemoresistance in OS are urgently needed.

Aberrant epigenetic reprogramming plays a pivotal role in tumorigenesis [7]. Among epigenetic regulation factors, histone methyltransferases (HMTs) are frequently dysregulated in a spectrum of human tumors, which indicates that HMTs are potential therapeutic targets [8]. Histone H3 lysine 27 trimethylation (H3K27me3) is frequently associated with gene repression and contributes considerably to tissue differentiation, stem cell properties, and cancer occurrence/progression [9]. Increases and decreases in H3K27me3 levels are catalyzed by the histone methyltransferase enzyme enhancer of zeste homolog 2 (EZH2) [10] and two demethylases belonging to the Jumonji C (JmjC) domain-containing family: ubiquitously transcribed tetratricopeptide repeat on chromosome X (UTX, also called lysine-specific demethylase 6A [KDM6A]) and Jumonji D3 (JMJD3, also called KDM6B) [11]. Thus far, increasing evidence has showed that dynamic methylation of H3K27 frequently occurs during the development of tumors, especially during the modulation of cancer stem cells (CSCs). H3K27me3 has been reported to be a negative modulator of breast CSCs (BCSCs), and upregulation of H3K27me3 content can suppress BCSCs [12]; the same phenomenon has also observed in ovarian CSCs [13] and in non-small cell lung cancer [14]. However, it remains unclear whether alterations in histone modifications such as H3K27me3 foster tumorigenesis and chemoresistance in OS.

Here, we illustrate that alterations in H3K27me3 are highly correlated with cisplatin sensitivity in OS and that increased H3K27me3 levels can enhance cisplatin efficacy against OS. This effect may be mediated by direct downregulation of PRKCA and MCL1 expression and subsequent inactivation of RAF/ERK/MAPK cascades as well as decreased phosphorylation of BCL2.

## Results

### Expression of KDM6A and KDM6B in OS cells and patient specimens

**Fig. 1:**
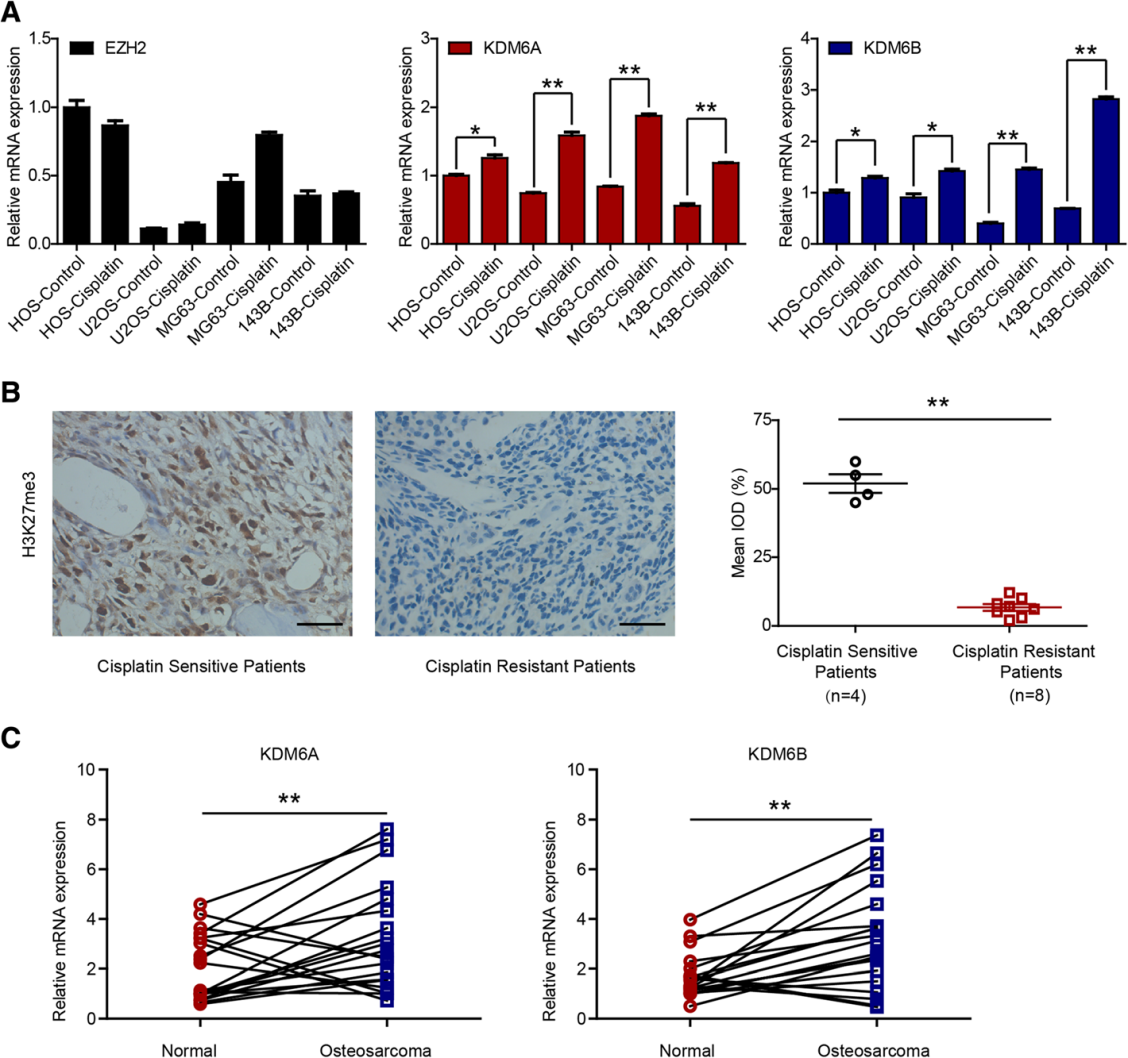
Expression of KDM6A and KDM6B in OS cells and patient specimens. **a** EZH2, KDM6A, and KDM6B mRNA levels in OS cells with or without cisplatin. **b** Immunohistochemical examination of H3K27me3 in cisplatin-sensitive (*n* = 4) and cisplatin-resistant specimens (*n* = 8). **c** KDM6A and KDM6B mRNA levels in 20 matched tumoral and peritumoral tissues. *P < 0.05, **P < 0.01. Abbreviation: IOD, integrated optical density

To explore the possible role of epigenetic methyltransferases and demethylases in OS, we first assessed the mRNA expression of 20 methyltransferases in OS cells treated with or without cisplatin (0.5 μM) for 24 h (Fig. 1a and Additional file 1: Figure S1). As shown in Fig. 1a, KDM6A and KDM6B were significantly upregulated after cisplatin treatment, while EZH2 was not induced. Since KDM6A and KDM6B are both demethylases of H3K27me3 [11, 15–17], we further investigated whether H3K27me3 levels were correlated with cisplatin resistance. The results indicated that H3K27me3 levels were higher in cisplatin-sensitive patient specimens than in cisplatin-resistant ones (Fig. 1b). We also analyzed the mRNA expression of KDM6A and KDM6B in 20 matched osteosarcoma and peritumoral tissues; the results showed that compared with normal peritumoral tissue, OS tissue expressed higher levels of KDM6A and KDM6B (Fig. 1c). Taken together, the results suggest that the expression of the H3K27 demethylases KDM6A and KDM6B is elevated in OS and that higher H3K27me3 levels indicate better efficacy of cisplatin against OS.

### Downregulation of KDM6A and KDM6B sensitizes OS to cisplatin in vitro

**Fig. 2:**
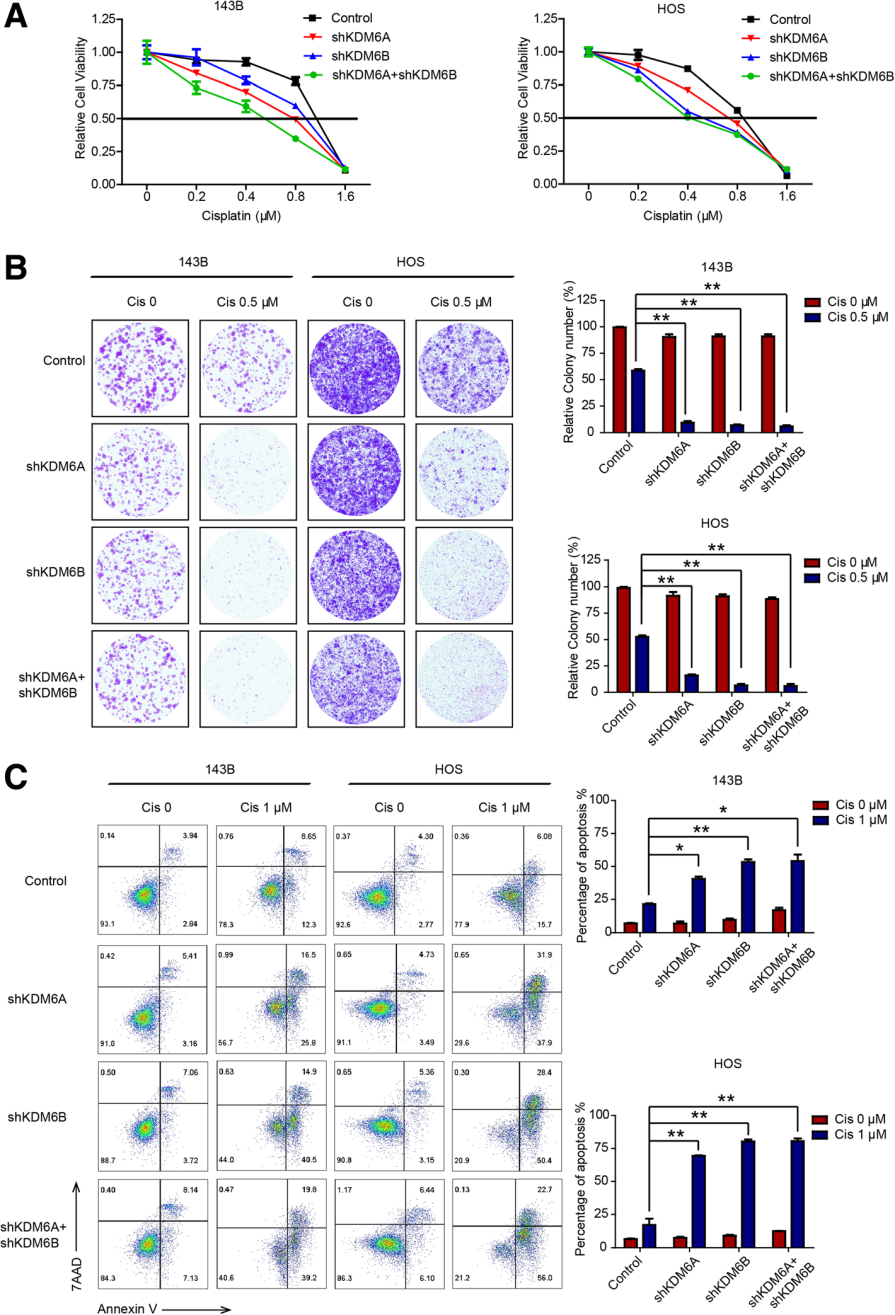
Downregulation of KDM6A and KDM6B sensitizes OS to cisplatin in vitro. **a** The viability of control and KDM6A or KDM6B-depleted 143B and HOS cells with or without cisplatin was tested by CCK-8 assay. **b** Colony formation of parental and KDM6A or KDM6B-depleted 143B and HOS cells with or without cisplatin. **c** The percentage of apoptotic cells among parental and KDM6A or KDM6B-depleted 143B and HOS cells with or without cisplatin was analyzed by flow cytometry. *P < 0.05, **P < 0.01

To further investigate the roles of KDM6A and KDM6B in OS, we established KDM6A- and KDM6B-depleted 143B and HOS cells and verified the success of knockdown by western blotting (Additional file 2: Figure S2). The results of Cell Counting Kit-8 (CCK-8) and colony formation assays showed that knockdown of KDM6A or KDM6B sensitized OS cells to cisplatin (Fig. 2a, b). The quantitative results revealed that the colony formation efficiency of KDM6A- and KDM6B-deficient cells decreased by 60–90% after cisplatin (0.5 μM) treatment. Additionally, the percentage of cells undergoing apoptosis was higher among KDM6A- and KDM6B-knockdown cells than among control cells, as revealed by Annexin V/7-aminoactinomycin D (7-AAD) staining (Fig. 2c). The quantitative results suggested there were four to sixfold increases in the percentage of apoptotic cells upon KDM6A or KDM6B knockdown. These observation were further confirmed by the western blot results, which showed higher cleaved Caspase 3 in KDM6A- and KDM6B-knockdown cells than in wild-type cells (Additional file 3: Figure S3). Collectively, our results indicated that KDM6A or KDM6B knockdown could sensitize OS to cisplatin by enhancing apoptosis of tumor cells.

### Increasing H3K27me3 levels sensitizes OS to cisplatin in vitro

**Fig. 3:**
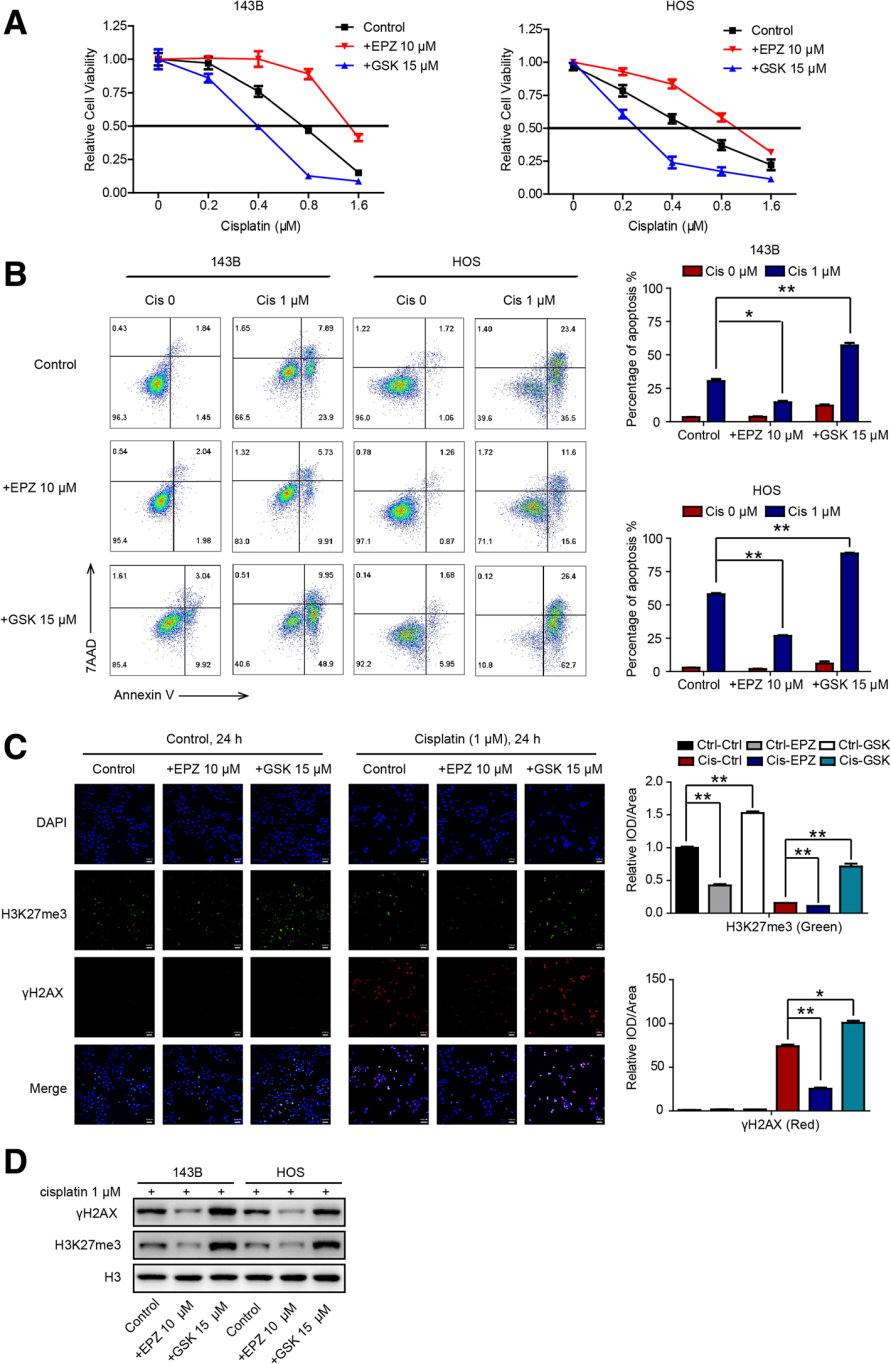
Increasing H3K27me3 levels sensitizes OS to cisplatin in vitro. **a** The viability of 143B and HOS cells without treatment and after treatment with EPZ (10 μM) or GSK-J4 (15 μM) for 72 h was analyzed by CCK-8 assay. **b** The percentage of apoptotic cells among 143B and HOS cells without treatment and after treatment with EPZ (10 μM) or GSK-J4 (15 μM) for 48 h was analyzed by flow cytometry. **c** Immunofluorescence of H3K27me3 (green) and γH2AX (red) in 143B cells subjected to the indicated treatments for 24 h. **d** H3K27me3 and γH2AX expression in OS cells subjected to the indicated treatments was analyzed by western blot analysis. * *P* < 0.05, **P < 0.01. Abbreviation: IOD, integrated optical density

KDM6A and KDM6B are the only two demethylases of H3K27me3 found so far [11], and KDM6A and KDM6B expression levels are associated with cisplatin sensitivity, as illustrated above. We subsequently verified the correlations between H3K27me3 levels and cisplatin resistance in OS. To achieve this, we used a selective inhibitor of KDM6A and KDM6B, GSK-J4 [18], and an inhibitor of EZH2 enzymatic activity, EPZ-6438 [19]. The results of CCK-8 and colony formation assays showed that increased H3K27me3 levels (GSK-J4 treatment) sensitized OS cells to cisplatin (Fig. 3a and Additional file 4: Figure S4). In addition, the percentage of cells undergoing apoptosis was significantly higher among high-level H3K27me3 cells treated with cisplatin than among low-level H3K27me3 cells treated with cisplatin (Fig. 3b). As it is known that the histone γH2AX is a marker for persistent DNA damage [20], we also monitored the efficacy of cisplatin against OS cells alone or in the presence of EPZ-6438 (10 μM) or GSK-J4 (15 μM) for 24 h by analyzing γH2AX foci. We found that compared to that in control cells, the number of γH2AX foci increased greatly after cisplatin treatment in OS cells, especially when they were cotreated with GSK-J4. In contrast, the number of foci decreased when cells were cotreated with EPZ-6438 (Fig. 3c). These results were further verified by western blot analysis, which showed higher γH2AX expression in OS cells cotreated with cisplatin and GSK-J4 and lower γH2AX expression in cells treated with cisplatin and EPZ-6438 than in control cells (Fig. 3d). Altogether, our results indicate that increasing H3K27me3 levels sensitizes OS to cisplatin in vitro.

### Upregulating H3K27me3 levels sensitizes OS to cisplatin in vivo

**Fig. 4:**
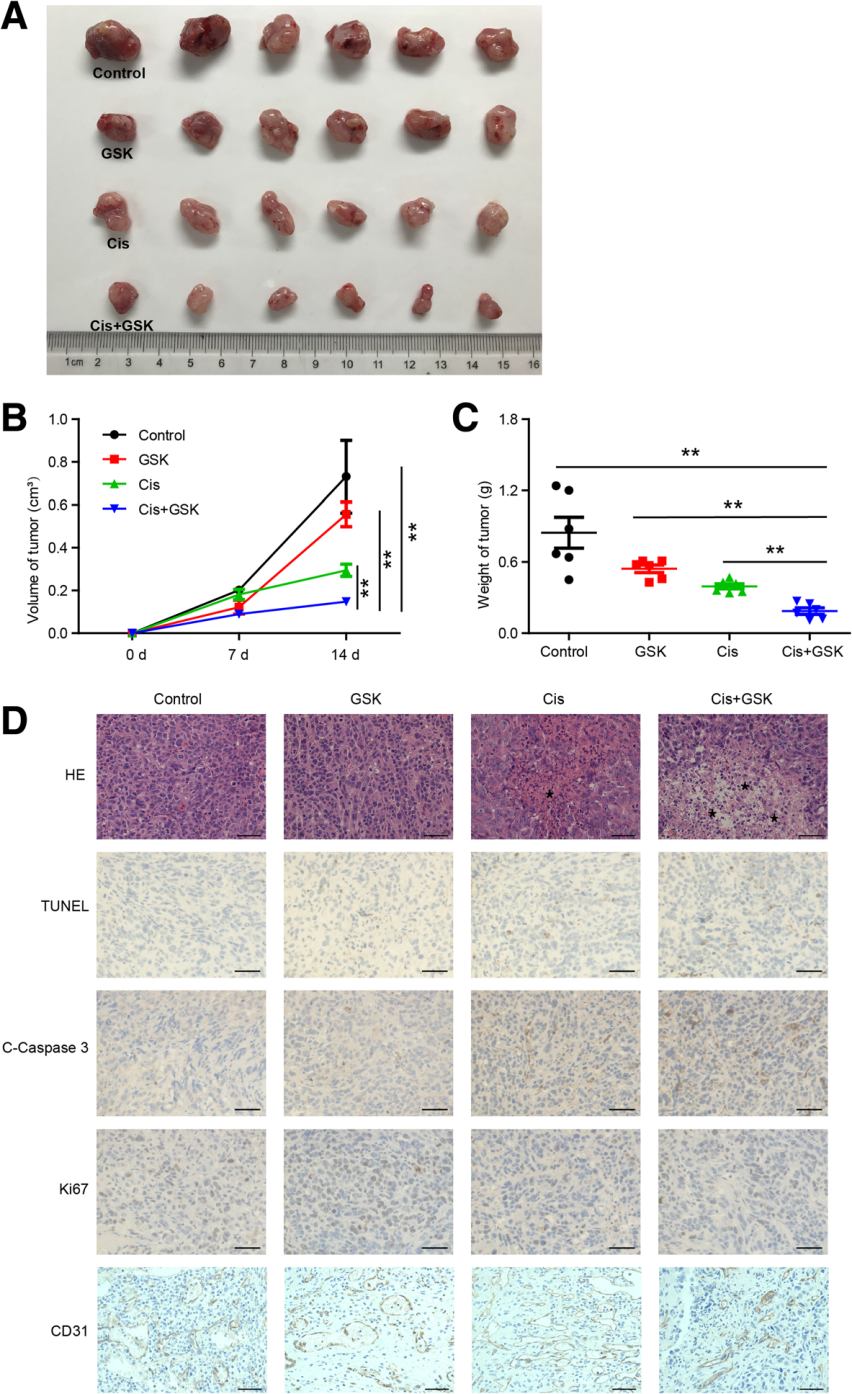
Upregulating H3K27me3 levels sensitizes OS to cisplatin in vivo. **a** Macroscopic image of tumor size after mice were sacrificed. Tumor volume (**b**) and weight (**c**) were measured at the indicated times. **d** H&E, TUNEL, cleaved Caspase 3, Ki67, and CD31 staining of representative tumors harvested from mice. **P < 0.01. Asterisk marks cell debris

We next determined the relationship between H3K27me3 and cisplatin efficacy in vivo. Twenty-four athymic nude mice received subaxillary injections of 143B cells. On day 5, we randomly divided the mice into four groups: the control (Ctrl) group, the GSK group, the Cis group, and the Cis+GSK group, which were treated with physiological saline, GSK-J4 (50 mg/kg intraperitoneally (ip) once per day (qd) beginning on day 5), cisplatin (8 mg/kg ip twice per week (biw) beginning on day 7), and GSK-J4 (50 mg/kg ip qd beginning on day 5) and cisplatin (8 mg/kg ip biw beginning on day 7), respectively. Most strikingly, there was a dramatic reduction in the size and weight of OS tumors in the group of mice cotreated with cisplatin and GSK-J4 compared with the other groups (Fig. 4a). On average, the tumor volumes and weights in the control group on autopsy were fourfold and threefold greater, respectively, than those in the cotreated group (Fig. 4b, c). Histopathological analysis showed that there was more cell debris (marked by asterisks in Fig. 4d first panel), an indicator of necrosis, in the Cis and Cis+GSK groups, especially in the cotreated group, than in the control group, as visualized by hematoxylin and eosin staining. We also assessed a range of markers in tumors by immunohistochemistry. The results showed that Ki67 expression was lower in the Cis and Cis+GSK groups, especially in the cotreated group, than in the control group, while the number of tumor cells positive for cleaved Caspase 3 or terminal deoxynucleotidyl transferase dUTP nick-end labeling (TUNEL) staining (apoptotic markers) was mostly elevated in the Cis+GSK group (Fig. 4d); there were no significant differences in the expression of CD31 (a marker of endothelial-like cells [21]) among the different groups. Taken together, these data support and verify the in vitro observations and demonstrate that high levels of H3K27me3 alleviate tumor malignancy and enhance apoptosis in combination with cisplatin in in vivo xenograft models, suggesting that H3K27me3 alterations are important in determining chemosensitivity in OS.

### H3K27me3 modification is associated with CSC properties in OS

**Fig. 5:**
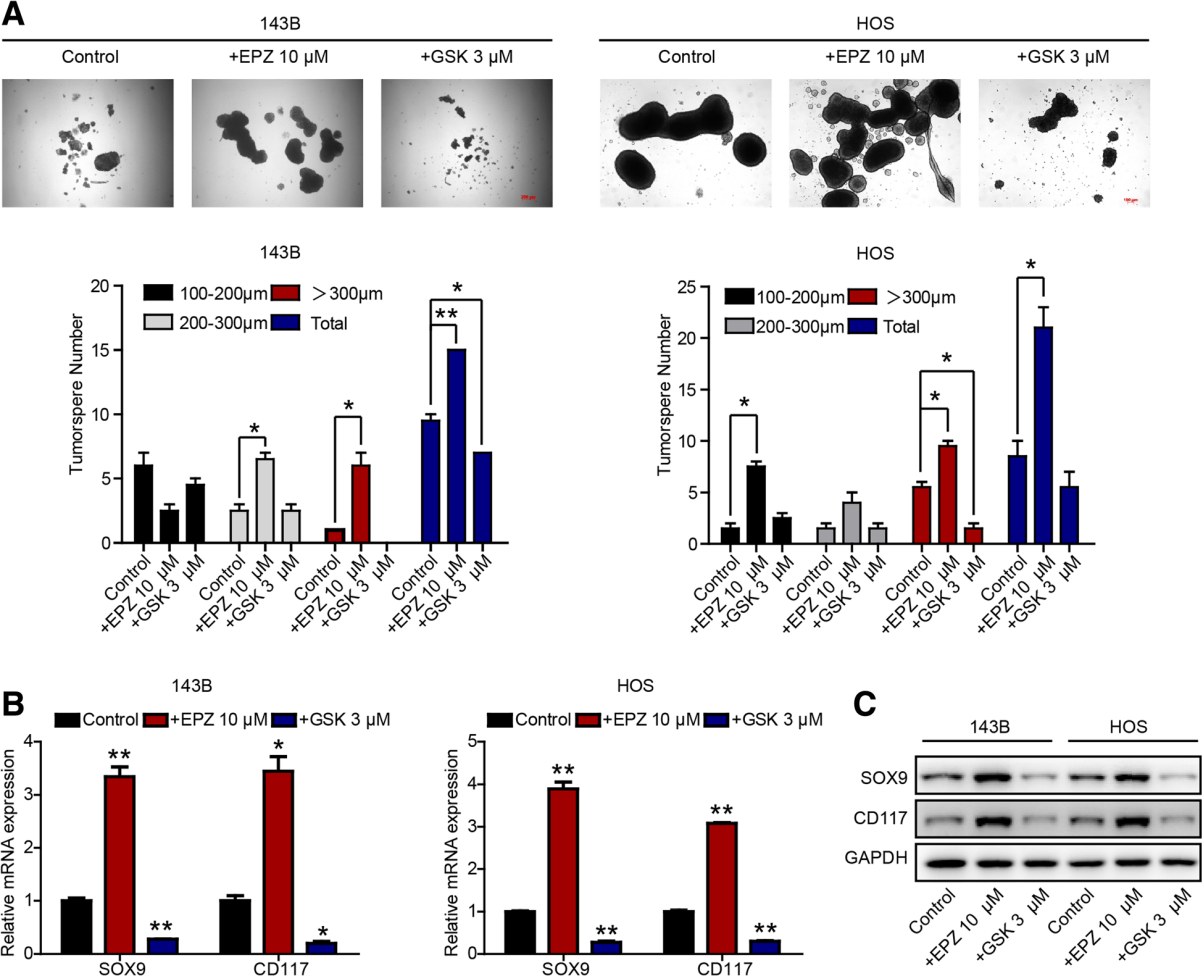
H3K27me3 modification is associated with CSC properties in OS. **a** Tumorsphere formation ability of OS cells subjected to the indicated treatments. SOX9 and CD117 expression in OS cells subjected to the indicated treatments was measured by RT-qPCR (**b**) and western blot analysis (**c**). *P < 0.05, **P < 0.01 compared with control

Cancer is widely believed to develop from mutant premalignant stem and progenitor cells. Accumulated mutations can confer further malignant properties, such as uncontrolled growth and proliferative advantages, on these cells, leading to tumorigenesis. Moreover, the marker H3K27me3 is closely associated with the state of stem cells, and KDM6A and KDM6B are highly expressed in embryonic stem cells [22]. Furthermore, there is a general assumption that CSCs, like their normal stem cell counterparts, are resistant to chemotherapy and/or radiotherapy and are therefore responsible for tumor relapse after therapy [23]. Therefore, we conducted further experiments to determine whether H3K27me3 levels influence CSC properties (stemness) in OS. We performed sphere formation experiments, since it is acknowledged that more spherical clones and larger spherical sizes indicate stronger self-renewal ability, which is a characteristic of CSCs. The results indicated that downregulating H3K27me3 with EPZ-6438 in HOS and 143B cells markedly increased the number and size of spheres, while upregulating H3K27me3 with GSK-J4 decreased the number and size of spheres (Fig. 5a). Furthermore, quantitative polymerase chain reaction (qPCR) and western blot results showed that the expression of both SOX9 and CD117 (which are associated with stem cell maintenance [24, 25]) increased when cells were exposed to EPZ-6438 but decreased when cells were exposed to GSK-J4 (Fig. 5b, c). Collectively, our data indicate that H3K27me3 modification is closely associated with CSC properties in OS and that higher H3K27me3 levels are correlated with reduced CSC properties and lower expression of CSC-related genes.

### Alterations in H3K27me3 regulate cell apoptosis- and stemness-related genes involved in OS chemosensitivity to cisplatin

**Fig. 6:**
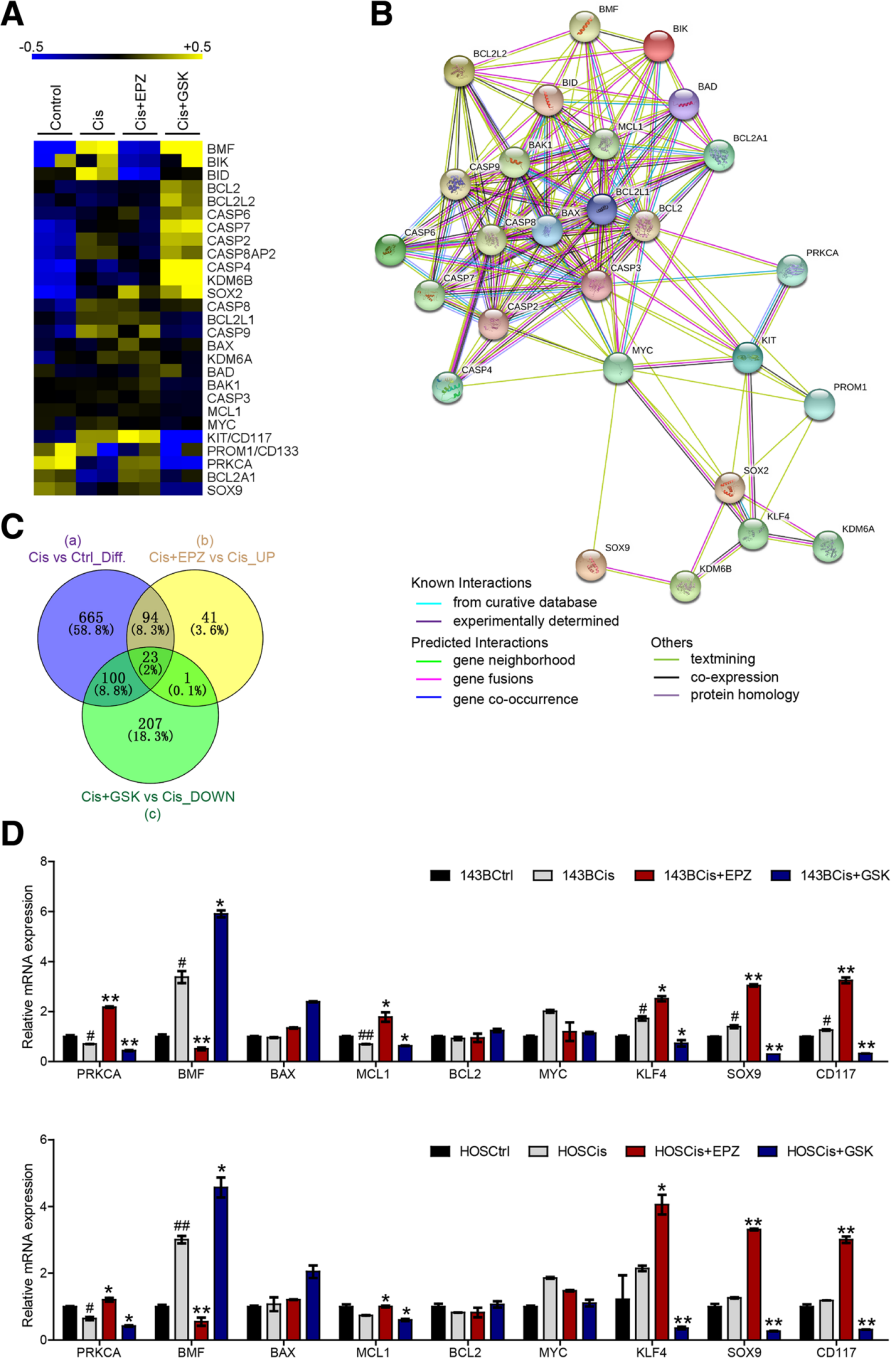
Alterations in H3K27me3 regulate apoptosis- and stemness-related genes involved in OS chemosensitivity to cisplatin. **a** Heatmap of differential gene expression profiles based on RNAseq. **b** Functional protein association network analysis of differential gene expression profiles based on RNAseq. **c** Venn diagram showing the number of differentially expressed genes in and shared between the indicated groups. **d** RT-qPCR analysis of *PRKCA, BMF, BAX, MCL1, BCL2, MYC, KLF4, SOX9,* and *CD117* expression. ^#^*P* < 0.05, ^##^*P* < 0.01 compared with the Ctrl group.*P < 0.05, **P < 0.01 compared with the Cis group

To gain insight into the mechanism by which higher H3K27me3 levels sensitize OS cells to cisplatin, we performed RNAseq on control (Ctrl), cisplatin-treated (Cis), cisplatin- and EPZ-6438-cotreated (Cis+EPZ), and cisplatin- and GSK-J4-cotreated (Cis+GSK) 143B cells. The results indicated that a number of gene expression profiles were changed by exposure to cisplatin with or without EPZ-6438 or GSK-J4, especially genes closely related to apoptosis and stem cell characteristics (Fig. 6a); these findings are similar to the results of a functional protein association network analysis [26] (Fig. 6b). To find the key molecules involved in this process, we mined the RNAseq data more deeply and discovered that 882 genes were differentially expressed in the Cis group compared with the Ctrl group (circle a), 159 genes were upregulated in the Cis+EPZ group compared with the Cis group (circle b), and 331 genes were downregulated in the Cis+GSK group compared with the Cis group (circle c). Interestingly, we found that the expression of *PRKCA*, one of 23 genes shared by circles a, b, and c, was changed significantly (Fig. 6c). Real-time (RT)-qPCR analysis further verified that higher H3K27me3 levels downregulated the expression of *PRKCA, MCL1, SOX9, CD117,* and *KLF4* but upregulated the expression of *BMF* (Fig. 6d). Altogether, our data revealed that alterations in H3K27me3 regulate apoptosis- and stemness-related genes involved in OS chemosensitivity to cisplatin.

### H3K27me3 alterations affect OS chemosensitivity through PRKCA via phosphorylation of BCL2 and modulation of RAF/ERK/MAPK cascades

**Fig. 7:**
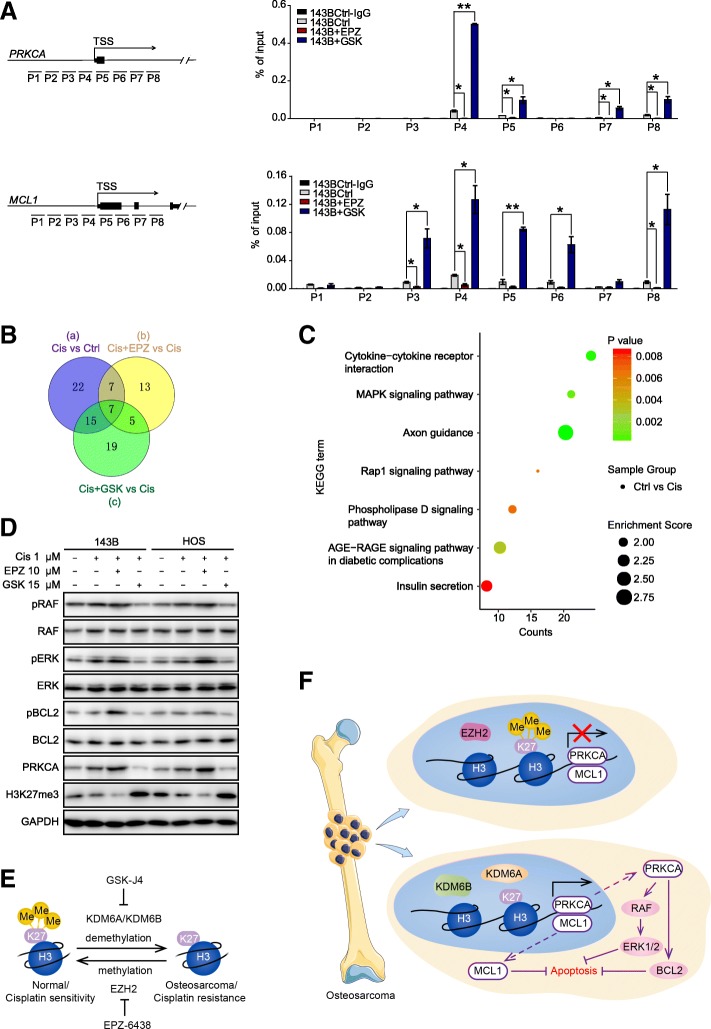
H3K27me3 alterations affect OS chemosensitivity through PRKCA via phosphorylation of BCL2 and modulation of RAF/ERK/MAPK cascades. **a** ChIP-qPCR analysis of H3K27me3 enrichment in the *PRKCA* and *MCL1* gene promoters in control, EPZ-6438-treated or GSK-J4-treated OS cells. **b** Venn diagram of the number of enriched signaling pathway in and shared between the indicated groups. **c** KEGG pathway analysis of 7 common overlapping pathways in the three circles in **b**. **d** Western blot analysis of pRAF, RAF, pERK, ERK, pBCL2, BCL2, PRKCA, and H3K27me3 levels (GAPDH serves as the control). **e** Schematic diagram of H3K27me3 alteration. **f** Schematic diagram of the underlying mechanism by which alterations in H3K27me3 affect OS chemosensitivity. Alterations in H3K27me3 directly regulate the expression of PRKCA and MCL1, leading to subsequent phosphorylation of BCL2 and activation of RAF/ERK/MAPK cascades. *P < 0.05, **P < 0.01

It is widely acknowledged that H3K27me3 methylation is associated with gene suppression, while demethylation of H3K27me3 is associated with gene activation. Since PRKCA, MCL1, SOX9, CD117, and KLF4 were upregulated in response to reduced H3K27me3 levels, we sought to determine whether the expression profile changes at these gene loci were directly associated with H3K27me3 alterations. Therefore, we conducted chromatin immunoprecipitation (ChIP)-qPCR, and the results showed that there were significant increases in H3K27me3 signals at the *PRKCA* and *MCL1* loci when cells were treated with GSK-J4; these results were reversed when cells were treated with EPZ-6438 (Fig. 7a). PRKCA, also known as protein kinase C alpha, has been reported to mediate a variety of cellular functions including proliferation, apoptosis, and differentiation [27–31]. In general, PRKCA exerts antiapoptotic effects and may serve as a survival factor in some types of cells [29]. In addition, in our study, overexpression of PRKCA in OS cells (PRKCA-OE cells), which was verified by western blot analysis (Additional file 5: Figure S5A), reversed the chemosensitization effects of GSK-J4 with regard to cisplatin, as demonstrated by flow cytometry (Additional file 5: Figure S5B). These findings provide further evidence for the role of PRKCA in mediating this process. Notably, the antiapoptotic effects of PRKCA have been shown to be mediated via activation of the RAF/ERK/MAPK cascade [32] and/or phosphorylation of the antiapoptotic protein BCL2 at serine 70 [33], and MCL1 is a member of the antiapoptotic BCL2 family that suppresses caspase activation [34]. In this study, Kyoto Encyclopedia of Genes and Genomes (KEGG) pathway analysis indicated that 51 pathway terms were associated with the genes differentially expressed between the Cis group and the Ctrl group (circle a), 32 pathway terms were associated with those between the Cis+EPZ group and the Cis group (circle b), and 46 pathway terms were associated with those between the Cis group and the Cis+GSK group (circle c) (Additional file 6: Table S5). In addition, seven terms were shared by circles a, b, and c (Fig. 7b), including the MAPK signaling pathway and the Rap1 signaling pathway (Fig. 7c). Based on the results mentioned above, we postulate that PRKCA regulates OS chemosensitivity by modulating BCL2 phosphorylation and the RAF/ERK/MAPK pathway. As expected, western blot analysis revealed that reductions in H3K27me3 levels were linked to upregulation of PRKCA, phosphorylated BCL2, phosphorylated RAF, and phosphorylated ERK, while total BCL2, RAF, and ERK remained stable (Fig. 7d). Taken together, our results illustrate that reductions in H3K27me3 levels induce OS chemoresistance via direct upregulation of PRKCA and MCL1 expression and that PRKCA subsequently phosphorylates BCL2 and activates RAF/ERK/MAPK cascades (Fig. 7e, f).

## Discussion

OS is a rare bone malignancy that primarily affects children and adolescents. At present, patients are typically treated with surgery and intensive adjuvant chemotherapy. However, unfortunately, the 5-year survival outcome for recurrent or metastatic OS is poor. Since the survival time of OS has not changed over the past two decades [35], new strategies and agents urgently need to be introduced to provide greater benefit to patients.

Epigenetic alterations are known to contribute greatly to carcinogenesis in humans [36]; therefore, epigenetic modulation could be a novel and promising strategy for fighting cancers, especially OS. Based on this possibility, we screened a panel of DNA methyltransferases and demethylases that could be induced by cisplatin in OS cell lines. The results indicated that KDM6A and KDM6B, which are both demethylases of H3K27, were upregulated by cisplatin treatment. We then sought to explore the correlations between H3K27me3 levels and chemosensitivity in OS and the underlying mechanism. The results indicated that the H3K27 demethylases KDM6A and KDM6B were upregulated in OS and that higher H3K27me3 levels indicated better efficacy of cisplatin against OS. In addition, KDM6A/KDM6B knockdown or upregulation of H3K27me3 levels sensitized OS to cisplatin by enhancing apoptosis in tumor cells.

It is natural to associate the link between epigenetic alterations and cancer chemosensitivity with stem cell properties, since epigenetic modification events are pivotal for stem cell renewal and differentiation and since CSCs are recognized to be more resistant than normal stem cells to chemotherapeutic agents [37]. Hence, in our study, we further tested CSC properties upon up- or downregulation of H3K27me3.

Our findings revealed that higher H3K27me3 levels were correlated with reduced CSC properties and reduced expression of CSC-related genes such as *SOX9* and *CD117*. Although we did not observe a significant change in the expression of SOX2 (a master regulator of stemness phenotype), we found that SOX9, which was also a SOX family member, showed a significant downregulation upon GSK-J4 treatment. SOX9 (SRY (sex-determining region Y)-box 9) is a member of the SOX family of transcription factors that plays critical roles in embryonic development, lineage commitment, and stem cell maintenance [38]. Likewise, CD117/c-kit is a tyrosine kinase receptor associated with cancer progression and normal stem cell maintenance [25], which also significantly downregulated upon GSK-J4 treatment.

In our study, RNAseq data indicated that *PRKCA*, *MCL1*, and other genes are also involved in this process. More importantly, through ChIP-qPCR analysis, we found that the expression of PRKCA and MCL1 was directly modulated by changes in H3K27me3 levels at the loci of these genes. The PKCs, which include PRKCA, are pivotal components of many signaling pathways that regulate diverse cellular functions including proliferation, angiogenesis, migration, invasion, metastasis, and resistance to apoptosis [39]. PRKCA (the α-isozyme of PKC) is widely expressed in various tissues, and abnormal levels of PRKCA have been found in many transformed cell lines and in several human tumors [40]. PRKCA has also been reported to be a dominant tumor progression factor that is associated with tumor proliferation and survival [41]. Pharmacological inhibition of PRKCA can reduce the growth and survival of tumors, promote apoptosis, and sensitize tumor cells to chemotherapy [42]. Mechanistically, the antiapoptotic action of PRKCA has been shown to be mediated by activation of RAF/ERK/MAPK cascades [43] and/or by phosphorylation of the antiapoptotic protein BCL2 [33]. Moreover, MCL1, is a member of the antiapoptotic BCL2 family, which can suppress apoptosis [34]. As the RAF/ERK/MAPK pathway is an important signaling pathway involved in cell survival, apoptosis inhibition, cell cycle progression, and proliferation, we reasonably associated PRKCA with the RAF/ERK/MAPK pathway and the phosphorylation of BCL2. Fortunately, in line with our hypothesis, RAF/ERK/MAPK pathway inactivation was found to participate in the process of OS sensitization to chemotherapy by increasing H3K27me3 levels on *PRKCA* and *MCL1* gene loci.

It remains unclear whether histone modification dynamics are the driving force of tumorigenesis and chemoresistance. PRKCA may also perform proapoptotic functions [27]; therefore, further investigation into the mechanism that influences the PRKCA functional mode is needed in the future. In addition, increasing numbers of studies have also demonstrated that epigenetic therapies enhance the efficacy of adoptive immunotherapies [44] and immune checkpoint inhibitors [45]. Thus, whether the immune system is involved in the process of chemosensitization also needs further exploration.

## Conclusions

In summary, we have shown, for the first time, that increasing H3K27me3 levels can promote the efficacy of cisplatin against OS by upregulating BMF and downregulating PRKCA, MCL1, SOX9, CD117, and KLF4, which subsequently dephosphorylates BCL2 and deactivates RAF/ERK/MAPK cascades. These findings may provide a promising strategy for enhancing chemotherapeutic efficacy against OS and improving prognoses of patients.

## Methods

### Tissue specimens

Patients with histologically confirmed OS who were involved in this study provided their informed consent, and all studies were performed following the guidelines of the ethics committee of Shanghai Ninth People’s Hospital. Twenty patients (six females and 14 males; there was no significant difference in composition regarding age or sex) were enrolled in this study (the clinical information is summarized in Additional file 6: Tables S1 and S2) and were divided into two groups based on their cisplatin efficacy outcomes: a cisplatin-sensitive group (cisplatin sensitivity 50–100%) and a cisplatin-resistant group (cisplatin sensitivity 0–50%).

### Immunohistochemistry

After deparaffinization, rehydration, antigen retrieval, and blockade of endogenous peroxidase, tissues were incubated with anti-TUNEL (Roche), anti-cleaved Caspase 3 (Servicebio), anti-Ki67 (Servicebio), and anti-CD31 (Abways) primary antibodies at 4 °C overnight. Next, secondary antibodies were applied, and diaminobenzidine (DAB) (Dako) solution was used as a chromogen. Finally, the sections were counterstained with hematoxylin (Sigma-Aldrich) to identify nuclei.

### Cell lines and cell culture

The human OS cell lines 143B and HOS were purchased from the Shanghai Institute of Biochemistry and Cell Biology (Shanghai, China). Cells were grown in Dulbecco’s modified Eagle’s medium (DMEM) (HyClone) supplemented with 10% fetal bovine serum (FBS; Gibco) and antibiotics (100 U/ml penicillin and 100 μg/ml streptomycin) at 37 °C in a humidified atmosphere with 5% CO_2_.

### Cell viability assay

A cell viability assay was performed as previously described [46]. Briefly, cells were added into 96-well plates at a density of 5 × 10^3^ cells/well. The cells were incubated with or without the indicated agents for 72 h, and then CCK-8 reagent was added into each well at a volume of 1:10 of that of the culture medium. The cells were then cultured for 2 h while protected from light. Data were collected by reading the optical density (OD) at 450 nm.

### RNA extraction and RT-qPCR

RNA extraction and RT-qPCR were performed as previously reported [47]. In brief, RNA was isolated from cell lines using TRIzol Reagent (Invitrogen), and RNA samples (1 μg) were subjected to RT-qPCR using an RT-PCR kit (Takara) according to the manufacturer’s protocols. Relative quantification (RQ) was calculated as RQ = 2^−ΔΔCt^. The sequences of the primers are listed in Additional file 6: Table S3.

### Protein extraction and western blot analysis

Cells were dissolved in lysis buffer containing protease inhibitors. Then, the proteins were subjected to 10% sodium dodecyl sulfate-polyacrylamide gel electrophoresis (SDS-PAGE) and transferred to 0.22 μm polyvinylidene fluoride (PVDF) membranes. The membranes were blocked with 5% nonfat milk at room temperature for 2 h and then incubated with primary antibodies, including antibodies against GAPDH (Cell Signaling Technology), H3K27me3 (Active Motif), H3 (Active Motif), PRKCA (Abways), BCL2 (Cell Signaling Technology), pBCL2 (Cell Signaling Technology), ERK (Cell Signaling Technology), pERK (Cell Signaling Technology), RAF (Abways), pRAF (Abways), γH2AX (Abways), SOX9 (Abways), CD117 (Abways), and cleaved Caspase3 (Abways), overnight at 4 °C. After three washes in Tris-buffered saline with Tween 20 (TBST), the membranes were incubated with corresponding secondary antibodies for 2 h at room temperature.

### Colony formation assay

Cells were seeded into six-well plates at a density of 1000 cells/well. After cell attachment, different agents were added. On day 7, the colonies were fixed and stained with 0.1% crystal violet. The number of colonies was counted manually.

### Flow cytometry analysis

A flow cytometry assay was performed as described previously [46]. In brief, the percentage of cells undergoing apoptosis was evaluated by flow cytometry (Beckman Gallios). Cells were incubated with different treatments for 48 h, harvested and resuspended in 500 μl of staining buffer with Annexin V-allophycocyanin (APC) and 7-AAD. The data were analyzed using FlowJo software.

### Tumorsphere formation assay

Single-cell suspensions were added to ultra-low-attachment multiwell plates (Costar) in serum-free chemically defined medium [48] with different treatments. Half of the volume was replaced with fresh medium every second day. Images were obtained on day 10.

### shRNA transfection and lentivirus packaging

KDM6A and KDM6B shRNA and the full protein-coding PRKCA open reading frame were synthesized by Sangon Biotech (Shanghai, China) and cloned into the pLVX plasmid (Open Biosystems) (sequences listed in Additional file 6: Table S4). Empty vectors were used as a negative control. Then, lentivirus was packaged using psPAX2 and pMD2G vectors. To obtain stable cell lines, lentivirus supernatant was added to the cells, and the cells were cultured with 2 μg/ml puromycin for 2 weeks.

### Xenograft experiments and histologic analysis

All animals were housed and maintained in specific pathogen-free (SPF) conditions. All animal experimental protocols were approved by the ethics committee of Shanghai Ninth People’s Hospital. Twenty-four athymic nude mice (6–8 weeks old) each were injected subcutaneously with 1 × 10^6^ 143B cells. On day 5 after injection, the mice were randomly divided into four groups (*n* = 6): the Ctrl group, the GSK group, the Cis group, and the Cis+GSK group. Beginning on day 5, the GSK group and the Cis+GSK group received ip injections of GSK-J4 (50 mg/kg) every day. Beginning on day 7, the Cis group and the Cis+GSK group received ip injections of cisplatin (8 mg/kg biw). The volume of each tumor was measured as (length × width^2^/2) weekly after implantation until the mice were sacrificed.

### Immunofluorescence

Cells were fixed and stained with antibodies against γH2AX (Abways) or H3K27me3 (Active Motif) after being treated with different agents for 24 h. After being washed in phosphate-buffered saline (PBS) three times, the cells were stained with the antibodies Alexa Fluor 555 (Invitrogen) and Alexa Fluor 488 (Invitrogen) for 1 h at room temperature. Images were obtained with a Cell Observer (ZEISS).

### RNAseq and KEGG analysis

RNA samples were obtained from control (Ctrl), cisplatin-treated (Cis) (cisplatin 1 μM, 24 h), cisplatin- and EPZ-6438-cotreated (Cis+EPZ) (cisplatin 1 μM, 24 h and EPZ-6438 10 μM, 24 h), and cisplatin- and GSK-J4-cotreated (Cis+GSK) (cisplatin 1 μM, 24 h and GSK-J4 15 μM, 24 h) 143B cells. After the proper pretreatments, the samples were subjected to RNAseq on a HiSeq 2500 system by Oebiotech Co. Ltd. Gene expression levels were analyzed with the programs htseq-count [49] and Cufflinks [50]. All differentially expressed gene lists were generated with DESeq software [51].

### ChIP-qPCR

Control and EPZ-6438 or GSK-J4 treated 143B cells (2 × 10^7^) were crosslinked, lysed, and sheared using UCD-300 (Bioruptor) to ~ 200–700 base pairs in length. Then, ChIP was performed using an EZ ChIP kit (Millipore; 17–371) according to the manufacturer’s instructions, followed by quantification of ChIP-enriched DNA by RT-qPCR. The primer sequences are listed in Additional file 6: Table S3.

### Statistical analysis

Each sample was analyzed in triplicate, and the experiments were repeated three times. The mean, standard error of the mean (SEM), and *P* values based on two-tailed *t* tests were calculated with Excel (Microsoft). Differences were considered significant at *P* < 0.05 (*P < 0.05, **P < 0.01).

## Additional files

Additional file 1: **Figure S1.** mRNA expression of methyltransferases and demethylases in OS cells with or without cisplatin treatment. (TIF 1114 kb)

Additional file 2: **Figure S2.** Knockdown efficacy of KDM6A and KDM6B as measured by western blot analysis. (TIF 217 kb)

Additional file 3: **Figure S3.** Knockdown of KDM6A or KDM6B enhances cisplatin-induced apoptosis in OS cells. Cleaved Caspase 3 expression in KDM6A- or KDM6B-knockdown and control OS cells with cisplatin treatment. (TIF 147 kb)

Additional file 4: **Figure S4.** Upregulation of H3K27me3 levels sensitizes OS to cisplatin. Colony formation ability of OS cells subjected to the indicated treatments. *P < 0.05. (TIF 1853 kb)

Additional file 5: **Figure S5.** Overexpression of PRKCA reverses the chemosensitization effects of GSK-J4 with regards to cisplatin. (A) Overexpression efficacy of PRKCA as measured by western blot analysis. (B) Apoptosis in PRKCA-overexpressing 143B and HOS cells and their parental cells as determined by flow cytometry. *P < 0.05, **P < 0.01. (TIF 881 kb)

Additional file 6: Supplementary Tables. (DOC 138 kb)

## Abbreviations

H3K27me3: Histone H3 lysine 27 trimethylation
OS: Osteosarcoma
