# Supplementary figures and images for: Elevated H3K27me3 levels sensitize osteosarcoma to cisplatin
Source: Clin Epigenetics. 2019 Jan 16;11:8. doi: 10.1186/s13148-018-0605-x (PMC6335728; doi:10.1186/s13148-018-0605-x)

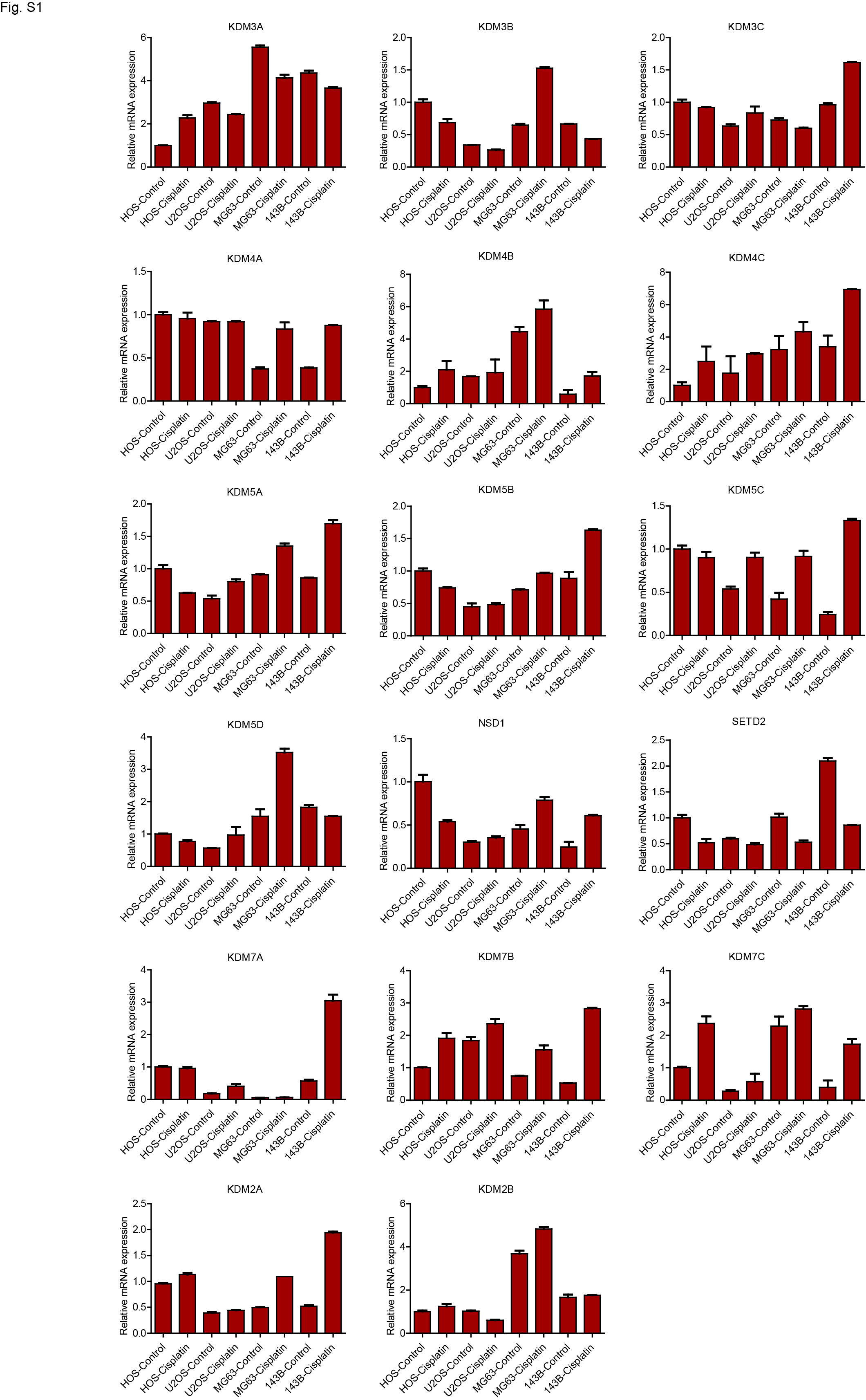

Supplement: Supplementary file 1 — Figure S1. mRNA expression of methyltransferases and demethylases in OS cells with or without cisplatin treatment. (TIF 1114 kb) [file 13148_2018_605_MOESM1_ESM.tif]

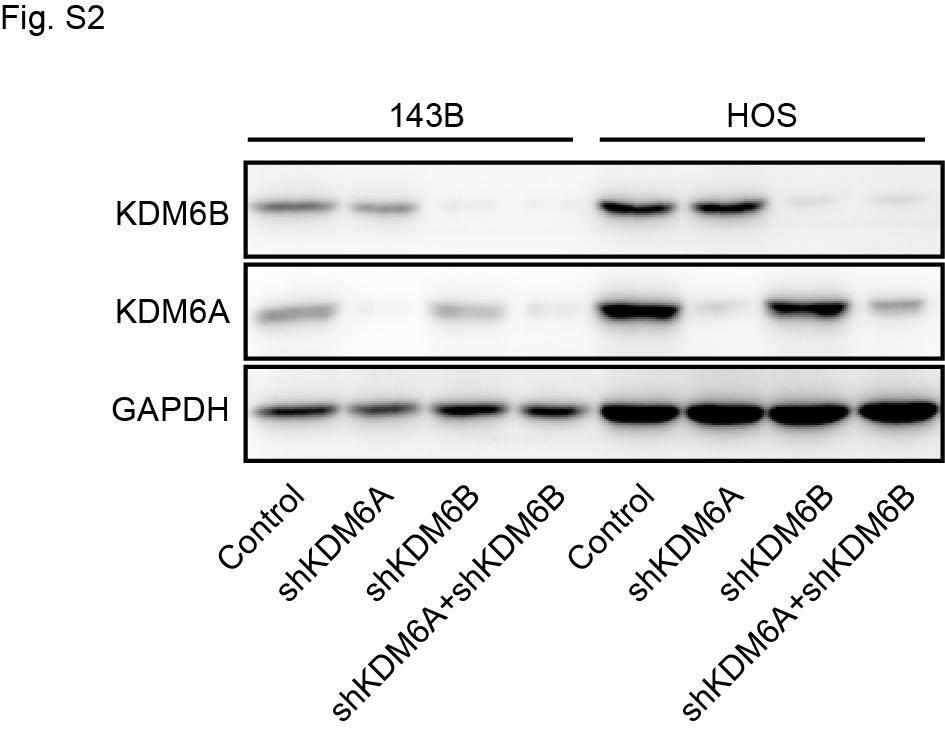

Supplement: Supplementary file 2 — Figure S2. Knockdown efficacy of KDM6A and KDM6B as measured by western blot analysis. (TIF 217 kb) [file 13148_2018_605_MOESM2_ESM.tif]

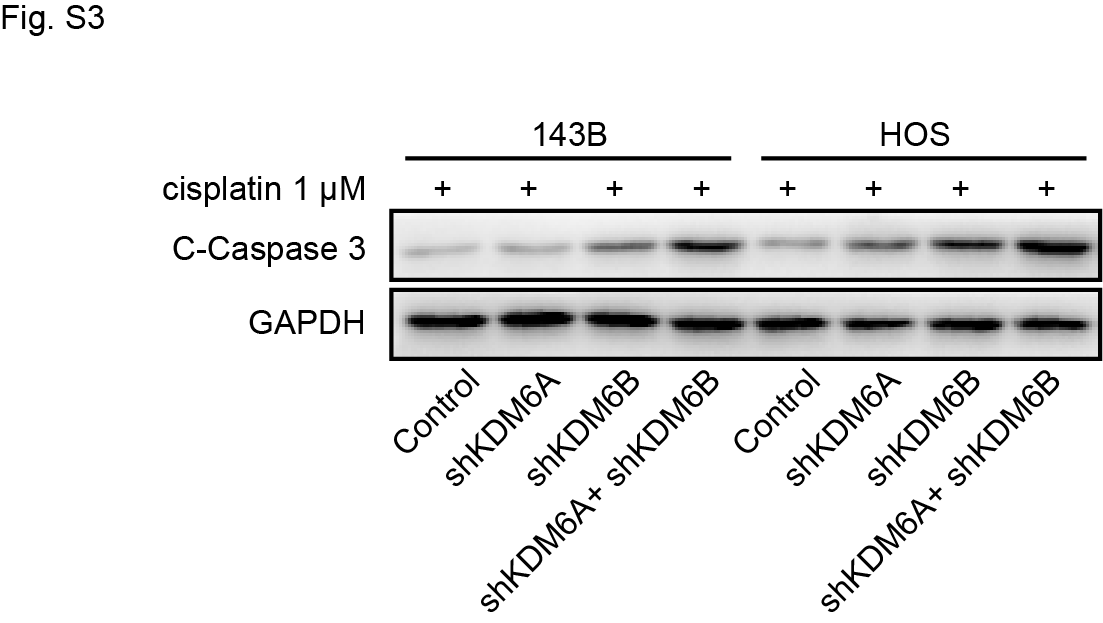

Supplement: Supplementary file 3 — Figure S3. Knockdown of KDM6A or KDM6B enhances cisplatin-induced apoptosis in OS cells. Cleaved Caspase 3 expression in KDM6A- or KDM6B-knockdown and control OS cells with cisplatin treatment. (TIF 147 kb) [file 13148_2018_605_MOESM3_ESM.tif]

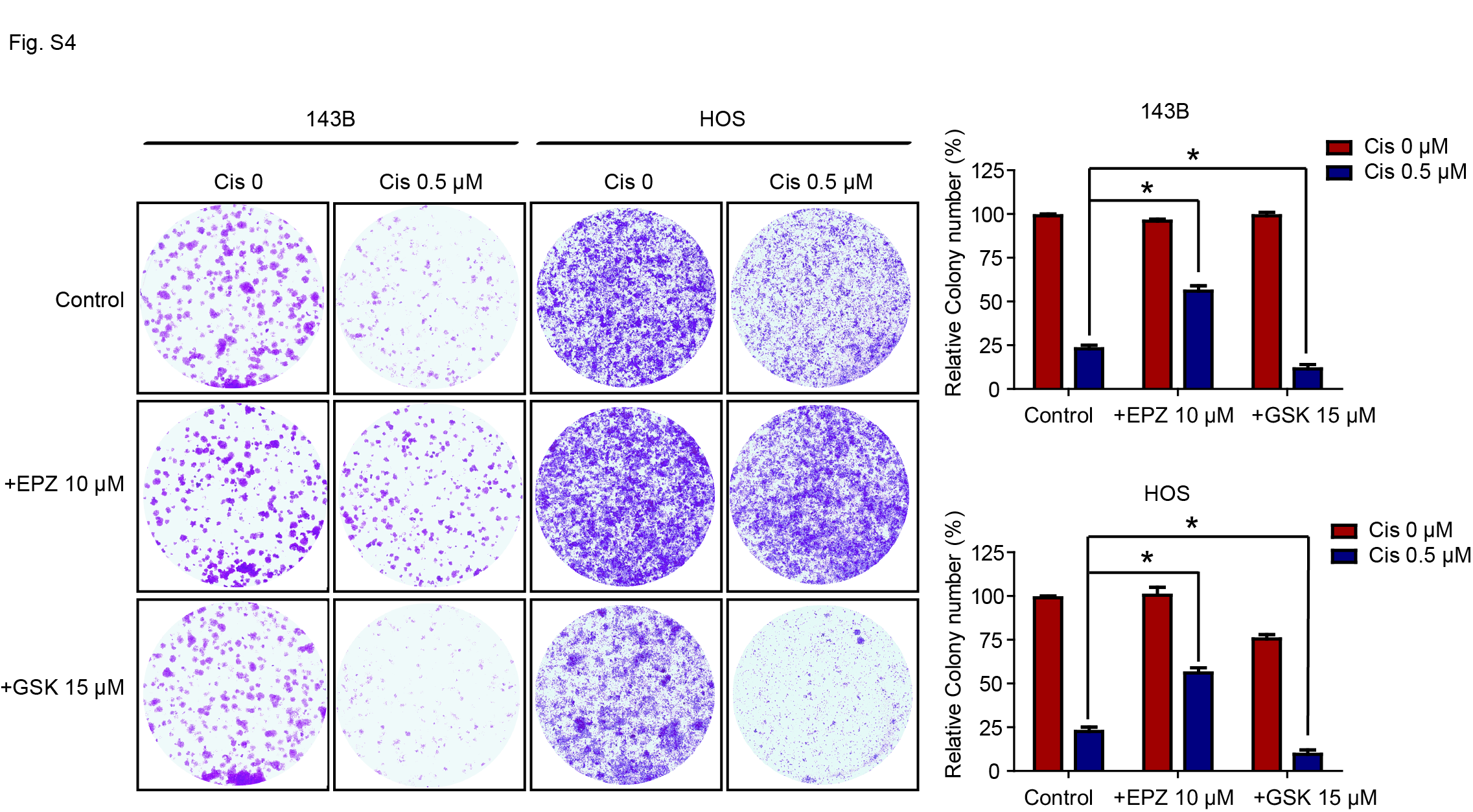

Supplement: Supplementary file 4 — Figure S4. Upregulation of H3K27me3 levels sensitizes OS to cisplatin. Colony formation ability of OS cells subjected to the indicated treatments. *P < 0.05. (TIF 1853 kb) [file 13148_2018_605_MOESM4_ESM.tif]

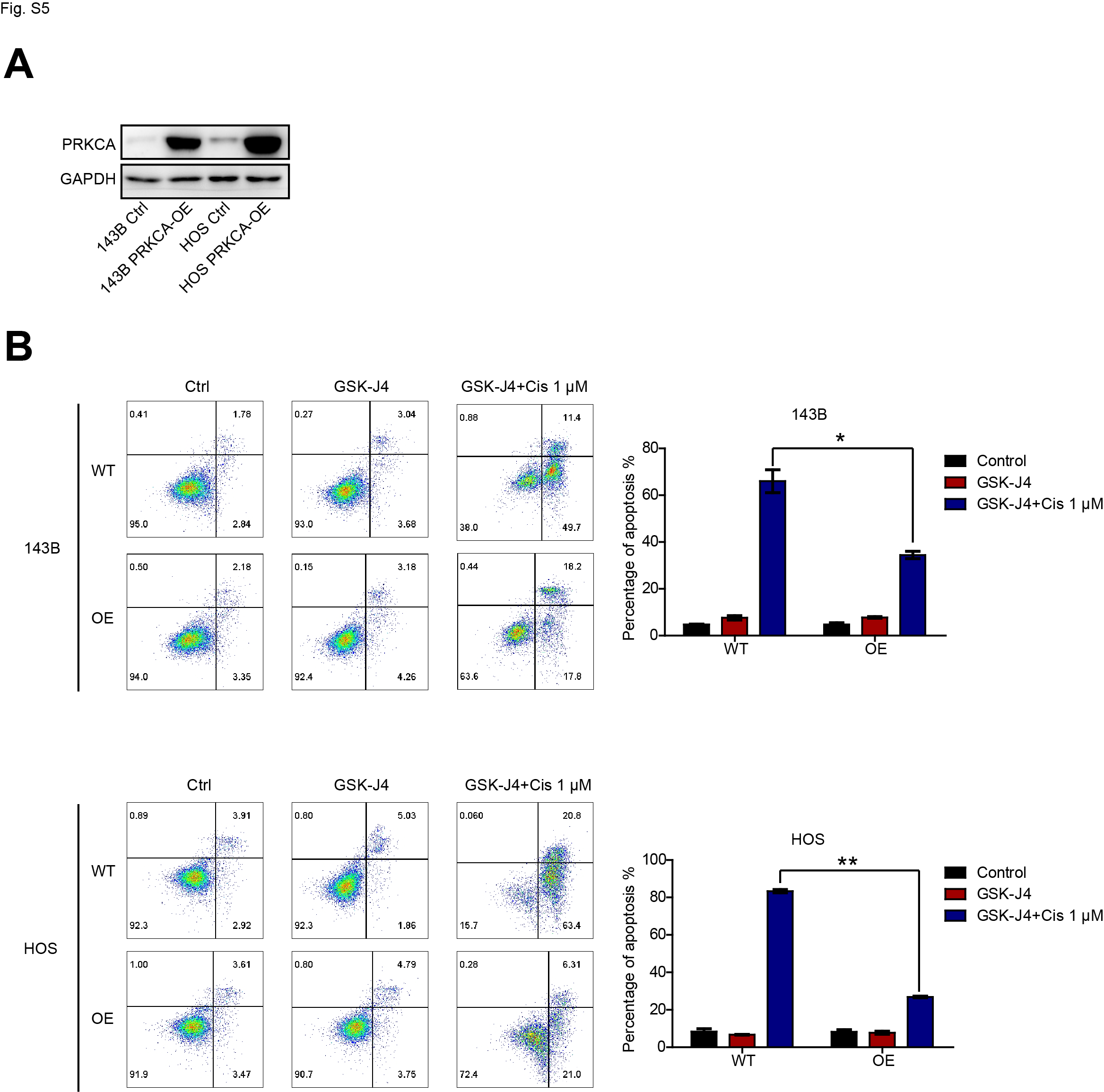

Supplement: Supplementary file 5 — Figure S5. Overexpression of PRKCA reverses the chemosensitization effects of GSK-J4 with regards to cisplatin. (A) Overexpression efficacy of PRKCA as measured by western blot analysis. (B) Apoptosis in PRKCA-overexpressing 143B and HOS cells and their parental cells as determined by flow cytometry. *P < 0.05, **P < 0.01. (TIF 881 kb) [file 13148_2018_605_MOESM5_ESM.tif]
