# Supplementary material for: Elevated H3K27me3 levels sensitize osteosarcoma to cisplatin
Source: Clin Epigenetics. 2019 Jan 16;11:8. doi: 10.1186/s13148-018-0605-x (PMC6335728; doi:10.1186/s13148-018-0605-x)
Supplement: Supplementary file 6 — Supplementary Tables. (DOC 138 kb) [file 13148_2018_605_MOESM6_ESM.doc]

**Additional file 6**

**Table S1.** **The** **clinical information of 20 paired osteosarcoma and peritumoral** specimens

| **Variables** | **All patients** |
| --- | --- |
| Numbers | 20 |
| Age at diagnosis, yr. | 10-78 |
| Year of surgery | 2006-2014 |

**Table S2.** **The clinical information of cisplatin sensitive (n=4) and resistant (n=8) specimens**

| **No.** | **Gender** | **Age** | **Pathology** | **Cisplatin Sensitivity** |
| --- | --- | --- | --- | --- |
| 1 | Male | 18 | OS | 54.28% |
| 2 | Male | 48 | OS | 85.58% |
| 3 | Male | 22 | OS | 58.50% |
| 4 | Male | 41 | OS | 83.68% |
| 5 | Female | 21 | OS | 1.91% |
| 6 | Female | 14 | OS | 10.39% |
| 7 | Male | 18 | OS | 20.13% |
| 8 | Male | 15 | OS | 0.00% |
| 9 | Male | 17 | OS | 22.97% |
| 10 | Male | 58 | OS | 37.16% |
| 11 | Male | 34 | OS | 9.42% |
| 12 | Male | 14 | OS | 8.19% |

Cisplatin Sensitivity 50%-100% defined as sensitivity; 0-50% defined as resistance. Abbreviation: OS, Osteosarcoma.

**Table S3. Primers of RT-qPCR**

| **RT-qPCR primers** | | |
| --- | --- | --- |
| **Gene name** | **Forward strand** | **Reverse strand** |
| KDM6A | GGACATGCTGTGTCACATCCT | CTCCTGTTGGTCTCATTTGGTG |
| KDM6B | ATCCGCGACCTCTGAACTCT | CGCTGCCTCACCCATATCC |
| EZH2 | AATCAGAGTACATGCGACTGAGA | GCTGTATCCTTCGCTGTTTCC |
| PRKCA | GTCCACAAGAGGTGCCATGAA | AAGGTGGGGCTTCCGTAAGT |
| BMF | CCAGAGCCTACTGGACTGC | AGCCAGCATTGCCATAAAAGA |
| BAX | CCCGAGAGGTCTTTTTCCGAG | CCAGCCCATGATGGTTCTGAT |
| MCL1 | TGCTTCGGAAACTGGACATCA | TAGCCACAAAGGCACCAAAAG |
| BCL2 | CATCCCAGCCTCCGTTAT | GTGGCCTTCTTTGAGTTCG |
| KLF4 | CGGACATCAACGACGTGAG | GACGCCTTCAGCACGAACT |
| MYC | GGCTCCTGGCAAAAGGTCA | CTGCGTAGTTGTGCTGATGT |
| SOX9 | AGCGAACGCACATCAAGAC | CTGTAGGCGATCTGTTGGGG |
| CD117 | CGTTCTGCTCCTACTGCTTCG | CCCACGCGGACTATTAAGTCT |
| GAPDH | CATCACGCCACAGTTTCC | ATCATCAGCAATGCCTCC |
| PRKCA-P1 | AAGGGAAGAAGCAAAGAG | CAAGCAATAAACCACCAT |
| PRKCA-P2 | CCAATGAAACGCTGAGAA | CATGGCTATGCAAAGGAC |
| PRKCA-P3 | TTCTCCTCCTCTTCTTCC | TGAAACCTCAAGCTGGTA |
| PRKCA-P4 | GCCCCAGGCTCACTGACA | CAGCACCGCAAGGCACTC |
| PRKCA-P5 | AAGGACCACAAATTCATC | CCAAGTTATCGGAGTGAG |
| PRKCA-P6 | AGGCTGGTCTCGAACTACTGAC | AGCAGGCAAATGGCACAA |
| PRKCA-P7 | TGAGACAGAGCGAGATTC | ATTCATTGGCGATTACCT |
| PRKCA-P8 | TGATGGATTCCGACAGAG | TTAGAGCAGCAGTAGTTTGG |
| MCL1-P1 | GCGAGTAGCGAGGAACAC | GGGATTATTGGCATGAGG |
| MCL1-P2 | CCAGATGGCCTGAAGTAA | TTGGGATAGGTGGTGAAG |
| MCL1-P3 | CTTGTAGTGAGCCGAGAT | TCCATAGTCTTTGGTGCT |
| MCL1-P4 | TCGTGGCTACCTCTGTGC | CCCCAACTATGCCCTCTT |
| MCL1-P5 | CTGCTCGCCACTTCTCAC | CCTTCTCCGTAGCCAAAA |
| MCL1-P6 | CCGTCCGTACTGGTGTTA | ATCATGTCGCCCGAAGAG |
| MCL1-P7 | GCTTCGGAAACTGGACAT | TTAGCCACAAAGGCACCA |
| MCL1-P8 | GGCACCGCCTTAGGAATT | ATGACGCACGGCTGTTTG |

**Table S4**. shRNA Sequences

| **KDM6A shRNA** | Top strand | gatcc**GCTGTTCGCTGCTATGAATTTCAAGAGAATTCATAGCAGCGAACAGC**TTTTTTACGCGTg |
| --- | --- | --- |
|  | Bottom strand | aattcACGCGTAAAAAA**GCTGTTCGCTGCTATGAATTCTCTTGAAATTCATAGCAGCGAACAGC**g |
| **KDM6B shRNA** | Top strand | gatcc**GATGATCTCTATGCATCCATTCAAGAGATGGATGCATAGAGATCATC**TTTTTTACGCGTg |
|  | Bottom strand | aattcACGCGTAAAAAA**GATGATCTCTATGCATCCATCTCTTGAATGGATGCATAGAGATCATC**g |

**Table S5**. KEGG pathway outcomes between indicated groups

| Cis vs Ctrl | | Cis+EPZ vs Cis | | Cis+GSK vs Cis | |
| --- | --- | --- | --- | --- | --- |
| id | term | id | term | id | term |
| path:hsa04360 | Axon guidance | path:hsa04360 | Axon guidance | path:hsa04360 | Axon guidance |
| path:hsa05202 | Transcriptional misregulation in cancers | path:hsa04540 | Gap junction | path:hsa04015 | Rap1 signaling pathway |
| path:hsa04115 | p53 signaling pathway | path:hsa04720 | Long-term potentiation | path:hsa04151 | PI3K-Akt signaling pathway |
| path:hsa04060 | Cytokine-cytokine receptor interaction | path:hsa04666 | Fc gamma R-mediated phagocytosis | path:hsa04014 | Ras signaling pathway |
| path:hsa04514 | Cell adhesion molecules (CAMs) | path:hsa05223 | Non-small cell lung cancer | path:hsa04916 | Melanogenesis |
| path:hsa04010 | MAPK signaling pathway | path:hsa04150 | mTOR signaling pathway | path:hsa05218 | Melanoma |
| path:hsa04971 | Gastric acid secretion | path:hsa04072 | Phospholipase D signaling pathway | path:hsa05205 | Proteoglycans in cancer |
| path:hsa04270 | Vascular smooth muscle contraction | path:hsa05231 | Choline metabolism in cancer | path:hsa00604 | Glycosphingolipid biosynthesis - ganglio series |
| path:hsa04933 | AGE-RAGE signaling pathway in diabetic complications | path:hsa05214 | Glioma | path:hsa04921 | Oxytocin signaling pathway |
| path:hsa04640 | Hematopoietic cell lineage | path:hsa04664 | Fc epsilon RI signaling pathway | path:hsa04924 | Renin secretion |
| path:hsa04727 | GABAergic synapse | path:hsa04060 | Cytokine-cytokine receptor interaction | path:hsa04270 | Vascular smooth muscle contraction |
| path:hsa05032 | Morphine addiction | path:hsa04520 | Adherens junction | path:hsa04350 | TGF-beta signaling pathway |
| path:hsa04015 | Rap1 signaling pathway | path:hsa04911 | Insulin secretion | path:hsa05200 | Pathways in cancer |
| path:hsa04072 | Phospholipase D signaling pathway | path:hsa04012 | ErbB signaling pathway | path:hsa04010 | MAPK signaling pathway |
| path:hsa04940 | Type I diabetes mellitus | path:hsa05215 | Prostate cancer | path:hsa04150 | mTOR signaling pathway |
| path:hsa04911 | Insulin secretion | path:hsa04650 | Natural killer cell mediated cytotoxicity | path:hsa04713 | Circadian entrainment |
| path:hsa04068 | FoxO signaling pathway | path:hsa04530 | Tight junction | path:hsa04720 | Long-term potentiation |
| path:hsa04670 | Leukocyte transendothelial migration | path:hsa05032 | Morphine addiction | path:hsa05031 | Amphetamine addiction |
| path:hsa04540 | Gap junction | path:hsa04514 | Cell adhesion molecules (CAMs) | path:hsa04933 | AGE-RAGE signaling pathway in diabetic complications |
| path:hsa04970 | Salivary secretion | path:hsa04510 | Focal adhesion | path:hsa04911 | Insulin secretion |
| path:hsa04974 | Protein digestion and absorption | path:hsa04916 | Melanogenesis | path:hsa04340 | Hedgehog signaling pathway |
| path:hsa04921 | Oxytocin signaling pathway | path:hsa04933 | AGE-RAGE signaling pathway in diabetic complications | path:hsa00260 | Glycine, serine and threonine metabolism |
| path:hsa05020 | Prion diseases | path:hsa04015 | Rap1 signaling pathway | path:hsa00562 | Inositol phosphate metabolism |
| path:hsa05143 | African trypanosomiasis | path:hsa04066 | HIF-1 signaling pathway | path:hsa04310 | Wnt signaling pathway |
| path:hsa05144 | Malaria | path:hsa04810 | Regulation of actin cytoskeleton | path:hsa04971 | Gastric acid secretion |
| path:hsa05030 | Cocaine addiction | path:hsa04630 | Jak-STAT signaling pathway | path:hsa04724 | Glutamatergic synapse |
| path:hsa04080 | Neuroactive ligand-receptor interaction | path:hsa04670 | Leukocyte transendothelial migration | path:hsa04068 | FoxO signaling pathway |
| path:hsa04924 | Renin secretion | path:hsa04722 | Neurotrophin signaling pathway | path:hsa04610 | Complement and coagulation cascades |
| path:hsa04725 | Cholinergic synapse | path:hsa04071 | Sphingolipid signaling pathway | path:hsa00410 | beta-Alanine metabolism |
| path:hsa04713 | Circadian entrainment | path:hsa05034 | Alcoholism | path:hsa04810 | Regulation of actin cytoskeleton |
| path:hsa04024 | cAMP signaling pathway | path:hsa04010 | MAPK signaling pathway | path:hsa04750 | Inflammatory mediator regulation of TRP channels |
| path:hsa04724 | Glutamatergic synapse | path:hsa04024 | cAMP signaling pathway | path:hsa00640 | Propanoate metabolism |
| path:hsa04512 | ECM-receptor interaction |  |  | path:hsa04925 | Aldosterone synthesis and secretion |
| path:hsa04915 | Estrogen signaling pathway |  |  | path:hsa04144 | Endocytosis |
| path:hsa04022 | cGMP - PKG signaling pathway |  |  | path:hsa04512 | ECM-receptor interaction |
| path:hsa04350 | TGF-beta signaling pathway |  |  | path:hsa05214 | Glioma |
| path:hsa05410 | Hypertrophic cardiomyopathy (HCM) |  |  | path:hsa04922 | Glucagon signaling pathway |
| path:hsa05134 | Legionellosis |  |  | path:hsa05030 | Cocaine addiction |
| path:hsa04630 | Jak-STAT signaling pathway |  |  | path:hsa05410 | Hypertrophic cardiomyopathy (HCM) |
| path:hsa05414 | Dilated cardiomyopathy (DCM) |  |  | path:hsa04060 | Cytokine-cytokine receptor interaction |
| path:hsa05412 | Arrhythmogenic right ventricular cardiomyopathy (ARVC) |  |  | path:hsa00250 | Alanine, aspartate and glutamate metabolism |
| path:hsa04912 | GnRH signaling pathway |  |  | path:hsa04072 | Phospholipase D signaling pathway |
| path:hsa04730 | Long-term depression |  |  | path:hsa04918 | Thyroid hormone synthesis |
| path:hsa00410 | beta-Alanine metabolism |  |  | path:hsa04261 | Adrenergic signaling in cardiomyocytes |
| path:hsa04710 | Circadian rhythm |  |  | path:hsa04974 | Protein digestion and absorption |
| path:hsa04510 | Focal adhesion |  |  | path:hsa04912 | GnRH signaling pathway |
| path:hsa04215 | Apoptosis - multiple species |  |  |  |  |
| path:hsa04726 | Serotonergic synapse |  |  |  |  |
| path:hsa04151 | PI3K-Akt signaling pathway |  |  |  |  |
| path:hsa04380 | Osteoclast differentiation |  |  |  |  |
| path:hsa04213 | Longevity regulating pathway - multiple species |  |  |  |  |
